# State-of-the-art liver disease research using liver-on-a-chip

**DOI:** 10.1186/s41232-022-00248-0

**Published:** 2022-12-09

**Authors:** Sayaka Deguchi, Kazuo Takayama

**Affiliations:** 1 Center for iPS Cell Research and Application (CiRA), Kyoto University, Kyoto, 606-8507 Japan; 2 Department of Medical Science, Graduate School of Medicine, Kyoto University, Kyoto, 606-8507 Japan; 3 AMED-CREST, Japan Agency for Medical Research and Development (AMED), Tokyo, 100-0004 Japan

**Keywords:** Organ-on-a-chip, Liver-on-a-chip, Hepatic zonation, Drug-induced liver injuries, Alcoholic liver diseases, NAFLD/NASH, Infectious liver diseases

## Abstract

To understand disease pathophysiologies, models that recapitulate human functions are necessary. In vitro models that consist of human cells are preferred to ones using animal cells, because organ functions can vary from species to species. However, conventional in vitro models do not recapitulate human organ functions well. Organ-on-a-chip technology provides a reliable in vitro model of the functional units of human organs. Organ-on-a-chip technology uses microfluidic devices and their accessories to impart organ functions to human cells. Using microfluidic devices, we can co-culture multiple cell types that compose human organs. Moreover, we can culture human cells under physiologically relevant stresses, such as mechanical and shear stresses. Current organ-on-a-chip technology can reproduce the functions of several organs including the liver. Because it is difficult to maintain the function of human hepatocytes, which are the gold standard of in vitro liver models, under conventional culture conditions, the application of liver-on-a-chips to liver disease research is expected. This review introduces the current status and future prospects of liver-on-a-chips in liver disease research.

## Background

Approximately 2 million people die of liver disease annually [1], and liver cirrhosis and liver cancer cause 3.5% of all deaths worldwide. The major causes of liver diseases are drug administration, alcohol intake, obesity, viral infection, and genetic mutations. Hepatitis is induced when the liver is damaged by these factors and risks liver fibrosis and cirrhosis. Moreover, patients with cirrhosis are more likely to develop liver cancer.

Several human liver models have been used to elucidate liver disease pathophysiology and to perform pharmaceutical research. Human hepatocytes isolated from cadaver donors and human liver chimeric mice, derived from human embryonic stem (ES) cells or induced pluripotent stem (iPS) cells, and hepatocellular carcinoma cell lines are widely used, as too more recently are liver organoids. Human hepatocytes isolated from cadaver donors and human liver chimeric mice lose their hepatic functions immediately after seeding on a two-dimensional (2D) cell culture plate. Additionally, their supply is limited. Human ES/iPS cell-derived hepatocyte-like cells and liver organoids, on the other hand, can form organ-like structures and have high hepatic functions, but they also lose their functions under conventional static 2D cell culture conditions. Some hepatocellular carcinoma cell lines, such as HepaRG cells, are known to possess high hepatic functions, but their characteristics are different from those of human liver. As a next generation model to reproduce liver functions, many researchers are attempting to culture hepatocytes and non-parenchymal cells in a dynamic 3D cell culture condition, for which organ-on-a-chip technology is attractive.

An organ-on-a-chip is an in vitro model that cultures human cells using microfluidic devices and their accessories. Using a microfluidic device, human cells can be arranged three-dimensionally to generate organ-like structures where the cells can be exposed to blood flow-induced shear stress by perfusing the cell culture medium. Moreover, human cells can be exposed to organ movement-induced mechanical stress by stretching the microfluidic device. Organ-on-a-chip technology is often used to recapitulate the functional units of organs, including the liver. In this review, we summarize the application of liver-on-a-chip technology for liver disease research.

## The structure and functions of human liver

The liver is an organ that plays an important role in xenobiotic metabolism and detoxification, bile acid synthesis, and immune responses [2–5]. Human liver is composed of parenchymal cells (hepatocytes) and non-parenchymal cells (liver sinusoidal cells (LSECs), hepatic stellate cells (HSCs), Kupffer cells (KCs), and cholangiocytes). Hepatocytes account for approximately 80% of liver cells, and non-parenchymal cells account for the remaining 20%. Hepatocytes play a central role in metabolism, detoxification, and bile acid synthesis. LSECs possess transcellular pores (fenestrae), form a permeable barrier [6] and are involved in exchanges between hepatocytes and sinusoidal blood. HSCs are present in the space between hepatocytes and LSECs. In liver diseases, HSCs take a myofibroblast-like phonotype and secrete fibrillar collagens [7]. KCs are macrophages located inside the liver sinusoid and are involved in the immune system [8]. Intrahepatic and extrahepatic bile ducts are composed of cholangiocytes. Bile acids synthesized in the liver pass through the bile ducts and are excreted into the duodenum. Taken together, hepatic functions are supported by all cells in the liver.

**Fig. 1:**
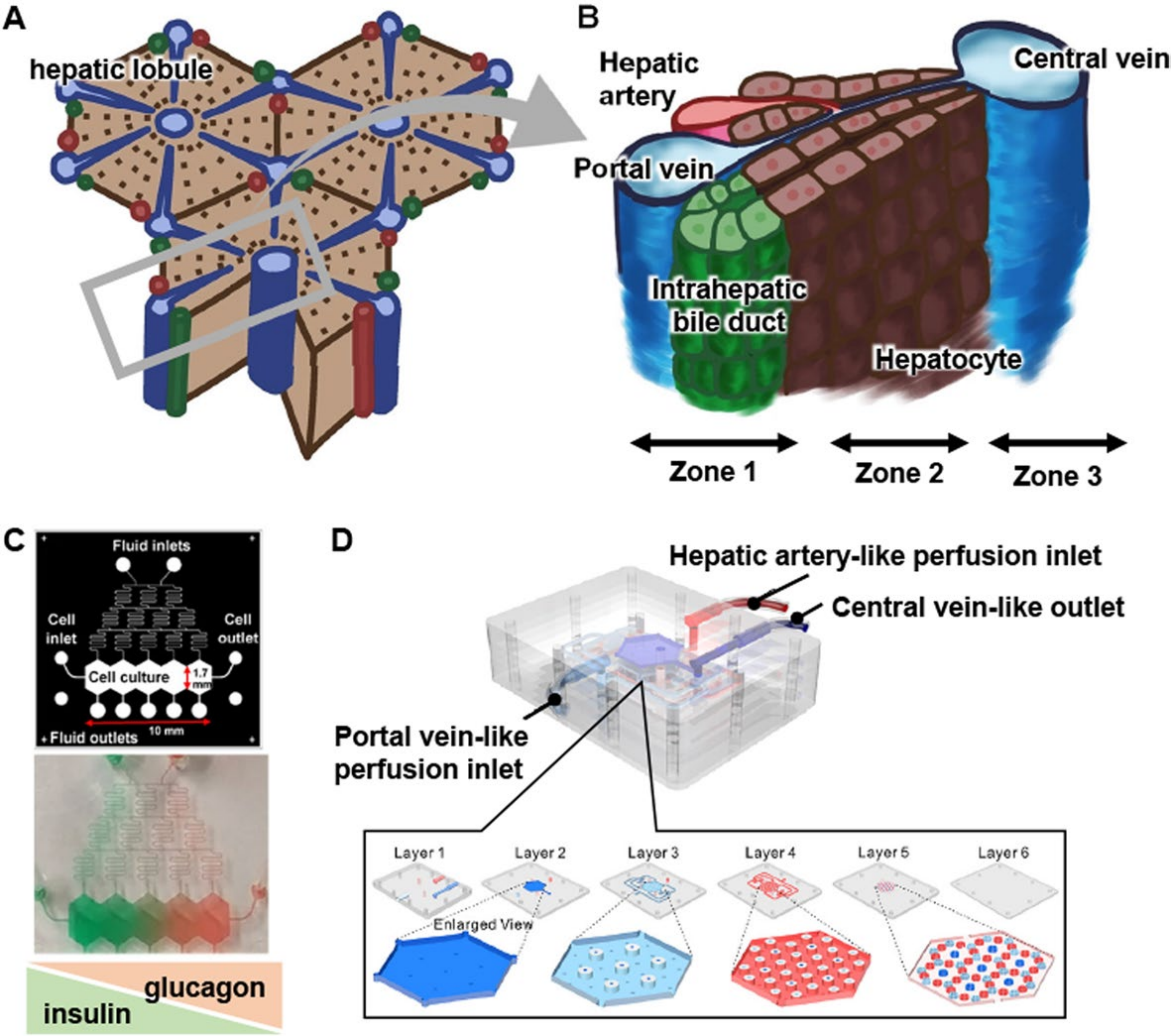
Recapitulation of hepatic zonation using liver-on-a-chips. **A** A schematic image of hepatic lobules. **B** A schematic image of the hepatic cord. **C** The design of metabolic patterning on a chip (MPOC) with a Christmas-tree-like gradient generator. Medium containing glucagon was injected into one of the inlets of the gradient generator, and medium containing insulin was injected into the other inlet. Adapted with permission under a Creative Commons CC BY 4.0 License from ref [9]. Copyright 2018 Springer Nature. **D** The design of a liver lobule chip (LLC). The LLC is composed of a portal vein- and hepatic artery-like perfusion inlet and central vein-like outlet. Reprinted with permission from ref [10]. Copyright 2021 American Chemical Society

Human liver is assembled by hepatic lobules (Fig. 1A), which are hexagonal in shape. The central vein is located at the center of the hepatic lobule, while the portal triad (portal vein, hepatic artery, and bile duct) is located at the corner. Plates of hepatocytes radiate out from the central vein to the portal triad and are called hepatic cords. Oxygen-rich arterial blood and nutrient-rich portal blood flow into the liver through the hepatic artery and portal vein, respectively, where they are mixed in the liver sinusoid. Oxygen and nutrients are consumed by the cells, and carbon dioxide and metabolites are excreted into the central vein blood.

The periportal region surrounding the portal triad is called “zone 1”, the pericentral region surrounding the central vein is called “zone 3”, and the midzonal region is called “zone 2” (Fig. 1B). Zone 1 hepatocytes are exposed to blood that contains oxygen or nutrients. On the other hand, zone 3 hepatocytes are exposed to blood that contains carbon dioxide and metabolites. It is known that the hepatic functions of hepatocytes differ depending on the zones. For example, mitochondrial β-oxidation and gluconeogenesis are higher in zone 1 hepatocytes, while lipogenesis and drug metabolism are higher in zone 3 hepatocytes [11–13].

## Recapitulation of hepatic zonation using liver-on-a-chip

In hepatic lobules, hepatocytes and non-parenchymal cells are robustly aligned to form hepatic zonation. Notably, it is difficult to recapitulate hepatic zonation only by culturing human hepatocytes under conventional static 2D cell culture conditions but less so with liver-on-a-chips.

First, we introduce liver-on-a-chips that reproduce the hepatic cord. The concentrations of oxygen and nutrients in zone 1 are higher than those in zone 3. McCarty et al. and Kang et al. developed metabolic patterning on a chip (MPOC) that consists of primary human or rat hepatocytes [9, 14]. Their microfluidic devices have a Christmas-tree-like gradient generator (Fig. 1C). Using this generator, they formed a substrate gradient in the cell culture chamber of their devices. Media containing glucagon or insulin were injected into separate inlets of the devices. By culturing hepatocytes under this condition, the hepatocytes could obtain the zonal activities of carbohydrate/glucose metabolism. Li et al. developed a vascularized human liver acinus microphysiological system (vLAMPS) that consists of primary human hepatocytes, primary LSECs, HSCs (LX-2 cells), and KCs (THP-1 cells) [15]. The upper hepatic channel contains human hepatocytes, HSCs, and KCs, and the lower vascular channel contains LSECs and KCs, and the channels are divided by a membrane. By perfusing the medium directionally, the oxygen concentration becomes high around the inlet of the hepatic channel (zone 1-like) and low around the outlet of the hepatic channel (zone 3-like). In addition, the mitochondrial membrane potential and lipogenesis are high in the zone 1-like and zone 3-like regions, respectively. Additionally, when HSCs are activated by pro-inflammatory mediators including LPS, TGF-β, and EGF, the expression levels of α-SMA (fibrosis marker) are increased more in the zone 3-like region than in the zone 1-like region.

Next, we introduce liver-on-a-chips that reproduce the hepatic lobe-like structure using hexagonal-patterned devices. Weng et al. developed a type of liver-on-a-chip they called LOC [16]. LOC consists of primary rat hepatocytes and HSCs and is connected to a peristaltic pump to circulate medium between the cell culture chamber and medium reservoir. This circulation mimics blood flow from the portal vein to the central vein. It is known that the drug-induced hepatotoxicity induced by acetaminophen (APAP) is more severe in zone 2 and 3 hepatocytes than zone 1 hepatocytes. In LOC, propidium iodide-positive dead cells are more frequently detected in the zone 2-like region than in the zone 1-like region. Ya et al. developed a liver lobule chip (LLC), which consists of primary mouse hepatocytes, LSECs, HSCs, and KCs (Fig. 1D) [10]. LLC is composed of portal vein- and hepatic artery-like perfusion inlets and a central vein-like perfusion outlet. In their LLC, LSECs self-assemble to generate a sinusoid network. Their LLC succeeded in forming an oxygen gradient that recapitulates the oxygen zonation in hepatic lobules. Other liver-on-a-chips have been developed to recapitulate the hepatic lobules: (1) the liver-cell patterning lab chip which patterns human hepatocytes (HepG2 cells) and endothelial cells (HUVECs) at the desired position using dielectrophoresis force [17]; (2) 3D liver lobule-like microtissue which is composed of radially patterned human hepatocytes (HepG2 cells) and endothelial cells (immortal human aortic endothelial cell line) [18]; (3) the liver-lobule-mimicking lab chip which is composed of radially patterned human hepatocytes (HepG2/ C3A cells) and fibroblasts (NIH/3T3 cells) [19].

Therefore, using organ-on-a-chip technology, hepatocytes and non-parenchymal cells can be cultured in an environment that reproduces hepatic lobules. Moreover, liver-on-a-chips with zone-specific hepatic properties can be used to study the heterogeneous pathophysiology of liver diseases.

## Liver disease research using liver-on-a-chips

In this section, we summarize studies using liver-on-a-chips to examine the pathophysiology of liver disease.

### Drug-induced liver injuries

**Fig. 2:**
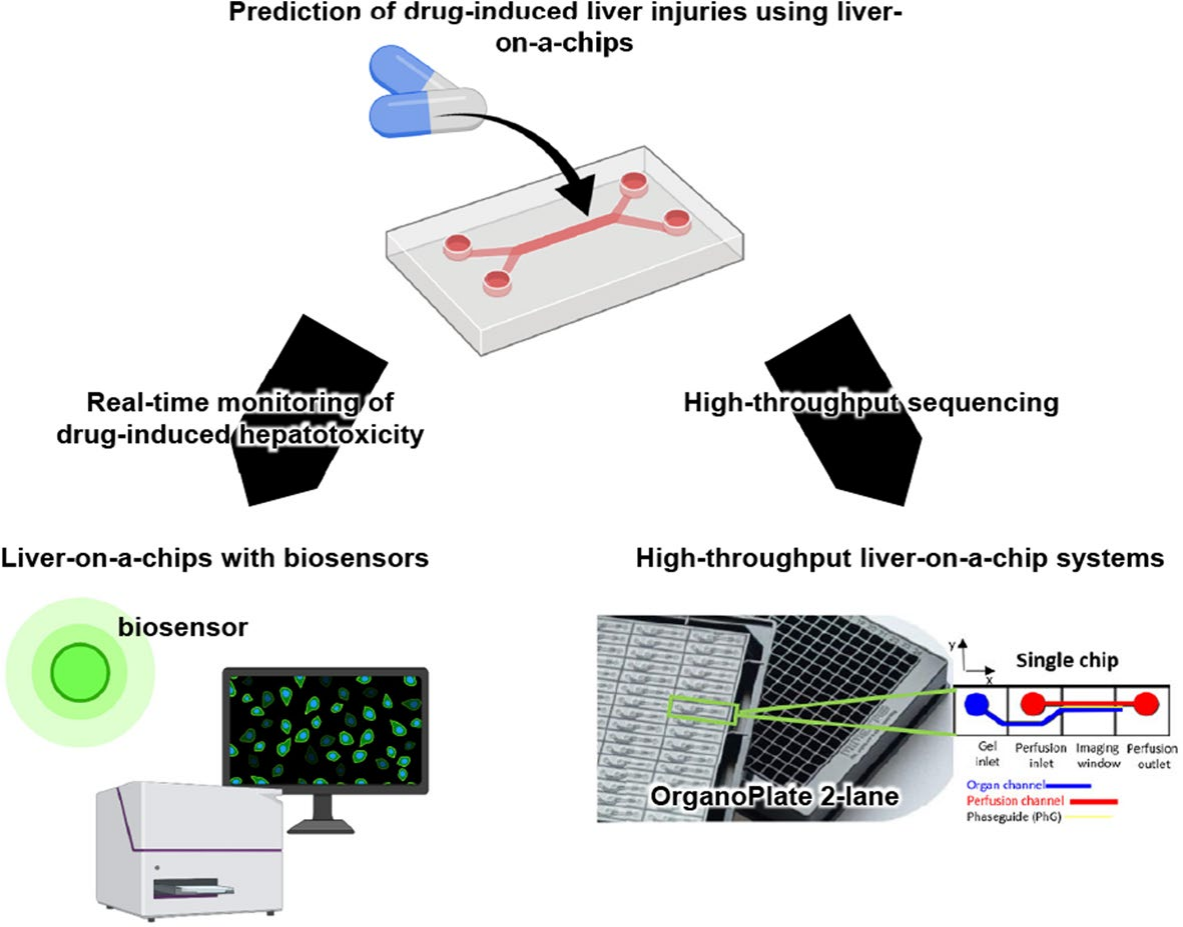
Study of drug-induced liver injuries using liver-on-a-chips. To perform real-time monitoring of drug-induced toxicities, liver-on-a-chips containing biosensors that detect cytotoxicity have been developed. To perform drug screenings, a high-throughput liver-on-a-chip system was generated. Created with Biorender.com. Adapted with permission under a Creative Commons CC BY 4.0 License from from ref [21]. Copyright 2021 Elsevier

Most drugs are metabolized by drug-metabolizing enzymes highly expressed in the liver, which makes the liver vulnerable to drug-induced liver injury [20]. It is difficult to evaluate these injuries in vitro, however, because the drug-metabolizing activities of hepatocytes cultured under conventional static 2D cell culture conditions are lower than those of human liver. In contrast, liver-on-a-chips are effective to maintain the drug-metabolizing activities of hepatocytes. In addition, because the exposure time before the onset of injury varies with the drug, liver-on-a-chips with biosensors, which can measure cytokine and oxygen concentrations, for real-time evaluation have been developed (Fig. 2).

Jang et al. developed Liver-Chip, which cultures primary human, rat, dog hepatocytes, LSECs, KCs, and HSCs in a microfluidic device with two parallel channels [22]. They confirmed that the transport activity of fluorescent bile salt derivatives (cholyl-L-lysyl-fluorescein and 5-(and-6)-carboxy-2′,7′-dichlorofluorescein diacetate) into the bile canaliculi was inhibited by the treatment of bosentan, a drug that is known to cause cholestasis. They also confirmed that oxidative stress was induced by APAP treatment, and liver steatosis was induced by methotrexate or fialuridine treatment. Notably, bosentan and fialuridine toxicity were observed in their Liver-Chip containing human cells but not rat cells, suggesting that species-specific drug-induced liver injuries could be evaluated.

Bavli et al. and Prill et al. developed liver-on-a-chips that culture oxygen sensor-embedded 3D aggregates of HepG2/C3A cells in a microwell bioreactor with medical sensors to measure glucose and lactate concentrations [23, 24] (Fig. 2, lower left). Bavli et al. found that a low concentration of troglitazone caused mitochondrial stress and induced a metabolic shift toward glycolysis, although the cell viability was unaffected [23]. Mitochondrial toxicity induced by APAP, amiodarone, valproate, or stavudine has been studied similarly [24, 25]. Vernetti et al. developed a 4-cell sequentially layered self-assembly liver model (SQL-SAL), which consists of human hepatocytes, endothelial cells (EA.hy926 cells), KCs (U937 cells), and HSCs (LX-2 cells) containing fluorescent protein biosensors [26]. SQL-SAL can perform a confocal high content analysis by monitoring the biosensor-labeled cells. They succeeded in evaluating mitochondrial dysfunction induced by troglitazone treatment, immune-mediated hepatotoxicity induced by trovafloxacin and lipopolysaccharide treatment, and hepatic fibrosis induced by methotrexate treatment.

To apply liver-on-a-chips for drug screening, it is necessary to develop a high-throughput liver-on-a-chip system. Khetani et al. developed the micropatterned co-culture (MPCC) platform, which consists of hepatocytes (primary rat, primary human, or human iPS cell-derived) and 3T3-J2 fibroblasts [27–30]. The albumin and urea secretion capacity and the drug-metabolizing ability of the hepatocytes were increased in this platform. Using a similar liver-on-a-chip, Ware et al. examined the toxicity of 47 drugs by measuring hepatic albumin, urea, and ATP levels [30]. A compound was considered “toxic” if the TC50 (median toxic dose) value was below 100*Cmax (maximum drug plasma concentration). The MPCC platform correctly classified 24 of 37 hepatotoxic drugs as “toxic” (65% sensitivity), and all 10 non-toxic drugs as “non-toxic” (100% specificity). Bircsak et al. developed the OrganoPlate LiverTox, which contains iPS cell-derived hepatocyte-like cells, endothelial cells (HMEC-1 cells), and KCs (THP-1) in an OrganoPlate 2-lane [21] (Fig. 2, lower right). The OrganoPlate 2-lane has 96 two-channels separated by a phase guide. The OrganoPlate LiverTox was treated with 159 compounds known to cause hepatotoxicity, and the Toxicity Priority Index score was calculated based on the cell viability, albumin secretion, urea secretion, and nuclear size.

Altogether, the above studies demonstrated that the risk of drug-induced liver injuries can be predicted accurately using liver-on-a-chips. Additionally, real-time monitoring and high throughput can be performed. With further development, liver-on-a-chips will contribute to the early prediction of drug-induced liver injuries and reduction of adverse drug events.

### Alcoholic liver diseases

**Fig. 3:**
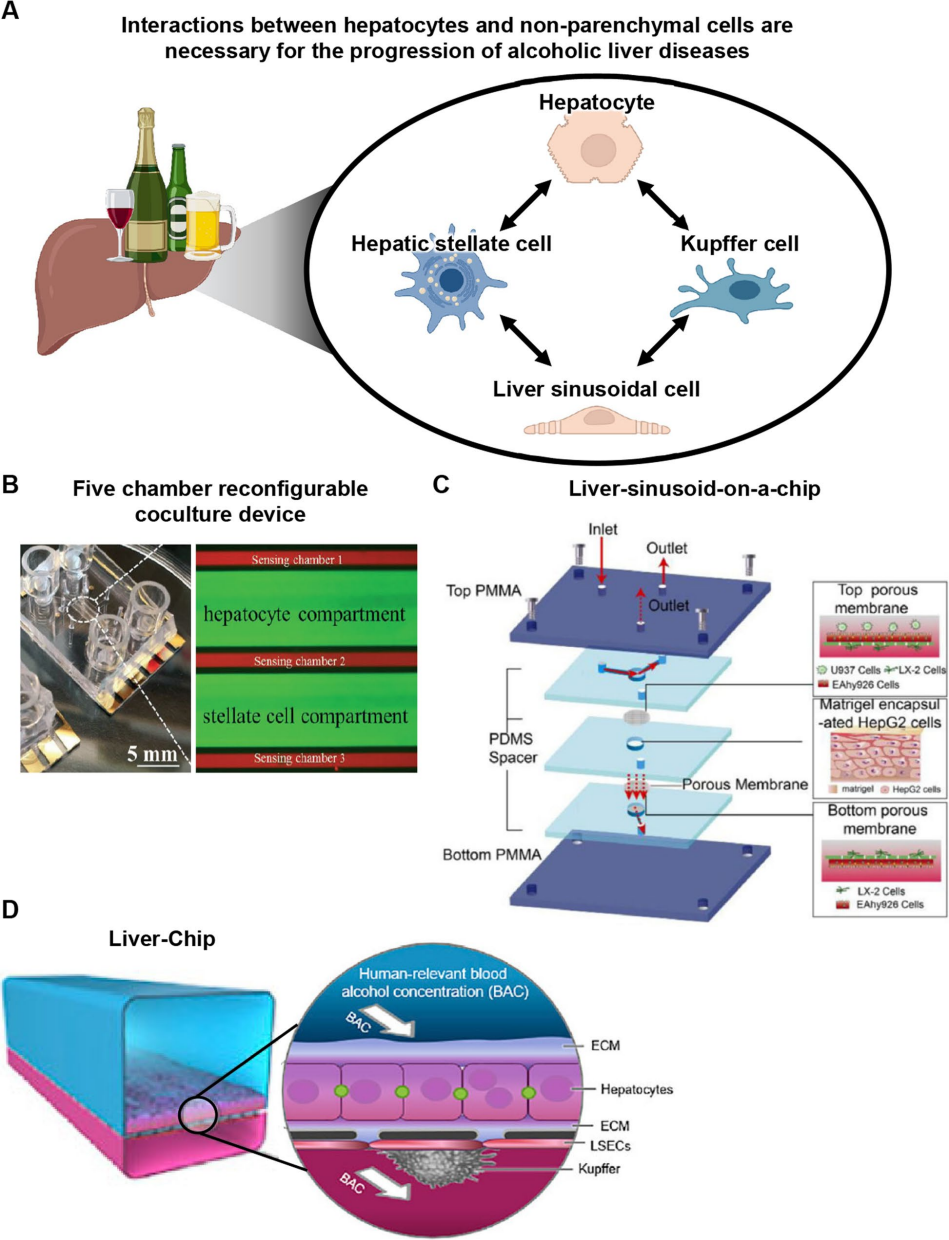
Study of alcoholic liver diseases using liver-on-a-chips. **A** Interactions between hepatocytes and non-parenchymal cells are essential for the progression of alcoholic liver disease. Created with Biorender.com. **B** The design of the 5-chamber reconfigurable coculture device, which can be utilized to evaluate interactions between hepatocytes and HSCs. Hepatocytes produce TGF-β after ethanol treatment and HSCs produce TGF-β due to exposure with TGF-β and other molecules secreted from injured hepatocytes. Reprinted with permission from ref [32]. Copyright 2015 Royal Society of Chemistry. **C** The design of the liver-sinusoid-on-a-chip, which can be utilized to evaluate interactions between hepatocytes, HSCs, and LSECs. By ethanol treatment, ROS production and α-SMA expression were enhanced in hepatocytes and HSCs, respectively. Reprinted with permission from ref [33]. Copyright 2019 Springer Nature. **D** The design of Liver-Chip, which can be utilized to evaluate interactions between hepatocytes, LSECs, and KCs. ROS production increases by ethanol, then normalizes after the recovery period. Adapted with permission under a Creative Commons CC BY-NC-ND 4.0 License from ref [34]. Copyright 2021 Elsevier

Excessive drinking can cause alcoholic liver diseases, which can lead to liver fibrosis or cirrhosis [31]. Liver steatosis and hepatic triglyceride accumulation are often observed during the early stages of alcoholic liver diseases. In the progression of alcoholic liver diseases, hepatocytes are damaged, and HSCs are activated (Fig. 3A).

Zhou et al. developed a liver-on-a-chip that cultures hepatocytes and HSCs in 5-chamber reconfigurable coculture device containing biosensors [32] (Fig. 3B). Two of the five chambers are cell compartments for culturing rat hepatocytes and HSCs (LX2 cells), and the other three have biosensors to monitor TGF-β secretion. To mimic drinking, medium containing ethanol is injected into the hepatocyte chamber. After that, the walls are raised to allow ethanol-injured hepatocytes to interact with HSCs, and the amount of TGF-β in the medium is measured by the biosensors. TGF-β production by the hepatocytes can be detected immediately after the ethanol treatment. HSCs also produce TGF-β due to exposure to TGF-β and other molecules secreted by the ethanol-injured hepatocytes. Deng et al. developed a liver-sinusoid-on-a-chip, which consists of human hepatocytes (HepG2 cells), LSECs (EAhy926 cells), HSCs (LX-2 cells), and KCs (U937 cells) [33] (Fig. 3C). They sandwiched hepatocytes between membranes whose two sides were seeded with LSECs and HSCs, respectively, and then injected KCs to mimic the liver sinusoid structure. Due to the ethanol exposure, ROS production in hepatocytes and vascular endothelial growth factor (VEGF) and α-smooth muscle actin (α-SMA) expression in HSCs were increased.

Alcohol abstinence can prevent the progression of alcoholic liver diseases [35]. Nawroth et al. developed another Liver-Chip, which contains human hepatocytes, LSECs, and KCs in a dual-channel microfluidic device with a porous membrane [34] (Fig. 3D). Human hepatocytes are cultured on the side of the porous membrane, while LSECs and KCs are cultured on the other side. Although ROS production was increased by ethanol, oxidative stress was normalized after the recovery period (5-day treatment of medium without ethanol). On the other hand, ROS production was not reduced after the recovery period in their Liver-Chip treated with ethanol and lipopolysaccharide (LPS) to mimic more severe alcoholic liver diseases. These results suggested that the effect of alcohol abstinence on alcoholic liver diseases of different severities can be clarified using liver-on-a-chips.

In summary, liver-on-a-chips can recapitulate invertible and irreversible lesions in alcoholic liver diseases caused by the interaction between hepatocytes and non-parenchymal cells. Therefore, liver-on-a-chips can help explore therapeutic targets and develop therapeutic drugs for these diseases.

### Non-alcoholic fatty liver disease/non-alcoholic steatohepatitis

**Fig. 4:**
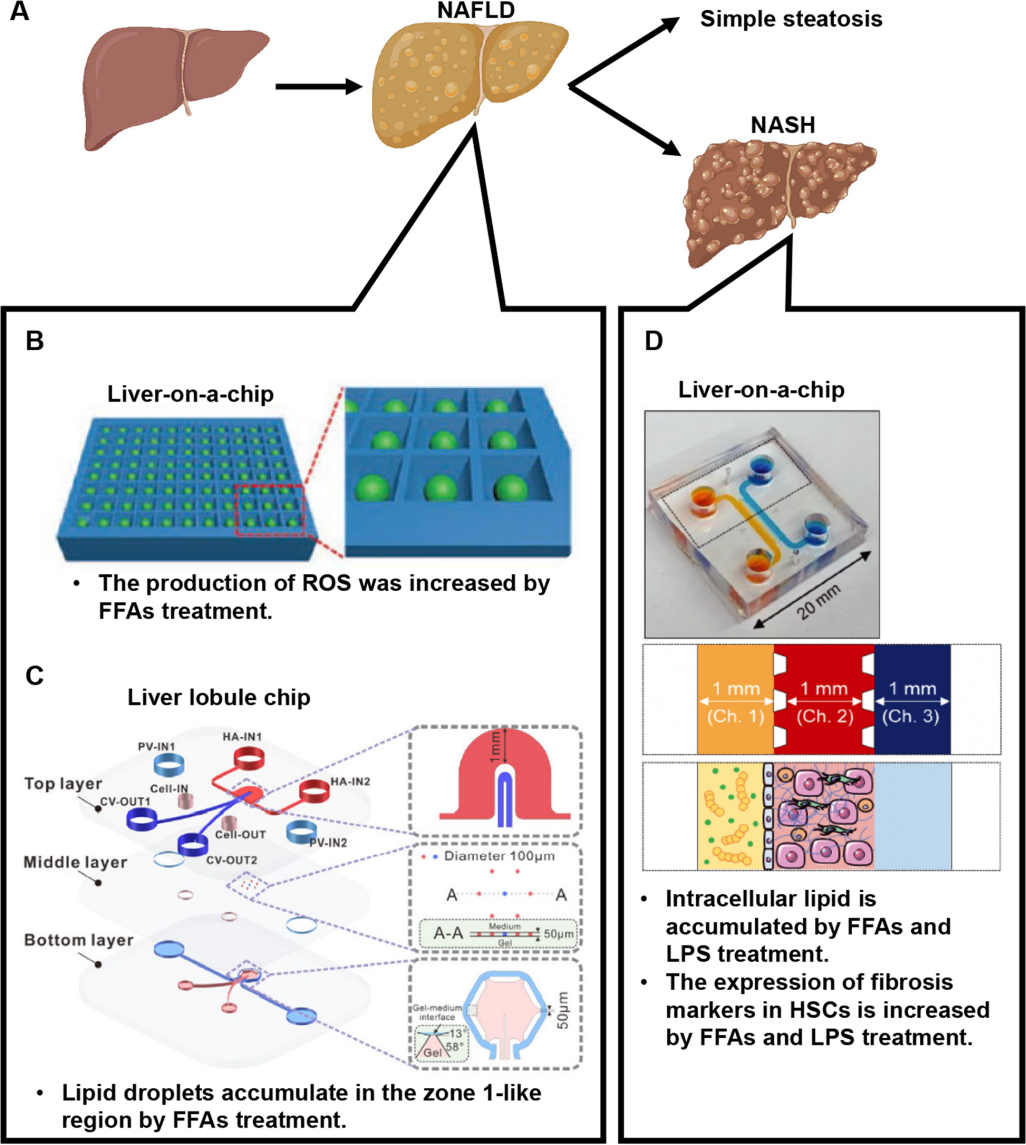
Study of non-alcoholic fatty liver disease/non-alcoholic steatohepatitis using liver-on-a-chip. **A** A schematic image of the progression of NAFLD/NASH. Created with Biorender.com. **B**, **C** To recapitulate liver steatosis observed in patients with NAFLD, a liver-on-a-chip (**B**) and liver lobule chip (**C**) were developed. Reprinted with permission from ref [38]. Copyright 2020 John Wiley and Sons. Reprinted with permission from ref [39]. Copyright 2021 Elsevier. **D** To recapitulate liver fibrosis observed in patients with NASH, a liver-on-a-chip was developed. Reprinted with permission from ref [40]. Copyright 2020 John Wiley and Sons

Non-alcoholic fatty liver disease (NAFLD) is liver steatosis that is not caused by alcohol or viral infection. There is a close association between obesity and NAFLD [36], and the number of NAFLD patients are increasing. NAFLD is classified based on clinical presentation into simple steatosis and non-alcoholic steatohepatitis (NASH) (Fig. 4A). Although the prognosis for simple steatosis is relatively good, inflammatory infiltration and fibrosis are often observed in patients with NASH [37]. The progression of NASH risks liver cirrhosis and liver cancer, and therapeutic drugs are needed.

Liver-on-a-chips have recapitulated liver steatosis in patients with NAFLD [41–44]. Lasli et al. developed a liver-on-a-chip that consists of human hepatocytes (HepG2 cells) and endothelial cells (HUVECs) [38] (Fig. 4B). Bioengineered liver tissue spheroids were generated using pyramid-shaped microwells and then transferred onto a networked microwell array. Medium containing two free fatty acids (FFAs), palmitic acid and oleic acid, were used to induce the steatosis and increased ROS production. The lipid accumulation in FFAs-treated liver-on-a-chips returned to basal level by treatment with metformin or pioglitazone, which are known antisteatotic drugs [45]. Du et al. developed a liver lobule chip, which consists of human hepatocytes (HepaRG cells), LSECs, and HSCs (LX2 cells) [39] (Fig. 4C). To mimic liver zonation, the medium was injected from the hepatic artery- and portal vein-like inlets and drained via the central vein-like outlets. The lipid accumulation was increased by adding lipogenic medium (glucose and FFAs). The amount of lipid droplets in the zone 1-like region was higher than that in the zone 2-like region after the treatment. Additionally, obeticholic acid and elafibranor, which are known to have beneficial effects on lipid metabolism, prevented or reversed the formation of lipid droplets [45–47].

In addition to steatosis, fibrosis progression is observed in patients with NASH. Freag et al. developed a liver-on-a-chip that contains human hepatocytes, HSCs, LSECs, and KCs in triplet microchannel devices [40] (Fig. 4D). Hepatocytes, HSCs, and KCs were injected into the middle channel, and LSECs were subsequently injected into the side channels of the device. To mimic the pathophysiology of NASH, lipotoxic medium containing palmitic acid, oleic acid, and LPS was infected into the endothelialized side channels. The intracellular lipid accumulation and the number of cleaved caspase-3-positive cells were increased under the lipotoxic conditions. Exposing liver-on-a-chips to lipotoxic stress also led to the development of NASH phenotypic characteristics, including hepatocellular ballooning and increased α-SMA (fibrosis markers) expression in HSCs, and increased TNF-α (inflammatory markers) secretion. Elafibranor, which is an effective NASH drug, was also tested [48, 49]. The therapeutic effects of many drug candidates for NAFLD/NASH are already being evaluated using liver-on-a-chips, including pioglitazon, metformin, obeticholic acid, elafibranor, pirfenidone, and ezetimibe [50–55].

Recently, it was reported that there is a strong correlation between the prognosis and genetic variants in NASH patients. Genome-wide association studies (GWAS) revealed that patatin-like phospholipase domain-containing protein 3 (PNPLA3) rs738409 C>G polymorphism (encoding I148M) is strongly associated with the severity of NAFLD/NASH [56]. Kostrzewski et al. developed a liver-on-a-chip to examine the effect of genetic polymorphisms in NASH [52]. They cultured human hepatocytes, KCs, and HSCs in perfused LiverChip platforms. In their model, the secretion of pro-inflammatory cytokines (IL-6 or TNF-α) and fibrosis markers (TIMP-1, fibronectin, and procollagen 1) was increased by FFAs treatment. To examine the effect of the PNPLA3 polymorphism, they generated a liver-on-a-chip consisting of hepatocytes, KCs, and HSCs carrying the PNPLA3 I148M mutation. IL-6 secretion in FFAs-treated liver-on-a-chips was enhanced by the presence of this mutation. This result suggests that liver-on-a-chips can be used to predict the effects of genetic polymorphisms related to NASH progression

### Infectious liver diseases

Various viruses, including hepatitis virus, cytomegalovirus, and herpes simplex virus, can infect human liver. The major cause of infectious liver diseases is hepatitis B virus (HBV) or hepatitis C virus (HCV) [57]. Because the development of therapeutic drugs for HBV has not progressed sufficiently, HBV studies using various liver models, such as human liver carcinoma cell lines including HepG2 or HuH7 cells, have been conducted. However, liver carcinoma cell lines do not faithfully reflect hepatic functions. Therefore, HBV studies using liver-on-a-chips are expected [58–61].

Kang et al. developed a liver-sinusoid-on-a-chip that contains human hepatocytes and endothelial cells (immortalized bovine aortic endothelial cells) in a dual microchannel with continuous medium perfusion [58]. After HBV infection, HBV DNA was detected in the cell culture supernatant, and approximately 72.9% of hepatocytes were positive for hepatitis B core Antigen (HBcAg). Ortega-Prieto et al. developed a liver-on-a-chip that contains human hepatocytes and KCs using the LiverChip platform to study HBV [60]. In a liver-on-a-chip with hepatocytes only, HBV infection did not induce an innate immune response, such as the phosphorylation of p38, JNK1/2/3, or STAT2. However, in a liver-on-a-chip with hepatocytes and KCs stimulated with LPS, HBV infection strongly induced the secretion of IL-6 and TNF-α, followed by a decrease of secretion of Hepatitis B surface antigen (HBsAg). This observation suggests that HBV infection can suppress innate immune activation in hepatocytes but not if in the presence of KCs. Overall, the results indicate that liver-on-a-chips can be utilized to clarify the contribution of hepatocytes and non-parenchymal cells to immune responses against a viral infection.

## Conclusions

Liver-on-a-chips are promising tools for recapitulating both the function and structure of human liver. The pathophysiology of drug-induced liver injuries, alcoholic liver diseases, NAFLD/NASH, and infectious liver diseases can be elucidated by exposing liver-on-a-chips to drugs, alcohol, FFAs, and hepatitis virus, respectively. Accordingly, liver-on-a-chips are already being used for liver disease and pharmaceutical research. By using iPS cell technology, it is possible to generate liver-on-a-chips that model specific genetic liver diseases. In addition, by connecting liver-on-a-chips with other organ-on-a-chips, organ-organ interactions can be analyzed. For example, developing liver-on-a-chips connected with bile ducts and intestinal tracts would allow us to better understand the interactions between the liver and these organs. With further developments, liver-on-a-chips will contribute to the development of therapeutic drugs for liver diseases for which there is no cure.

## Data Availability

Not applicable

## Abbreviations

2D: Two-dimensional
3D: Three-dimensional
APAP: Acetaminophen
ES: Embryonic stem
FFAs: Free fatty acids
GWAS: Genome-wide association studies
HBcAg: Hepatitis B core antigen
HBsAg: Hepatitis B surface antigen
HBV: Hepatitis B virus
HCV: Hepatitis C virus
HSCs: Hepatic stellate cells
iPS: Induced pluripotent stem
KCs: Kupffer cells
LLC: Liver lobule chip
LOC: Liver-on-a-chip
LPS: Lipopolysaccharide
LSECs: Liver sinusoidal cells
MPCC: Micropatterned co-culture
MPOC: Metabolic patterning on a chip
NAFLD: Non-alcoholic fatty liver disease
NASH: Non-alcoholic steatohepatitis
PNPLA3: Patatin-like phospholipase domain-containing protein 3
SQL-SAL: 4-cell sequentially layered self-assembly liver model
VEGF: Vascular endothelial growth factor
vLAMPS: Vascularized human liver acinus microphysiological system
α-SMA: α-smooth muscle actin
